# Blood–Brain Barrier Dysfunction and Astrocyte Senescence as Reciprocal Drivers of Neuropathology in Aging

**DOI:** 10.3390/ijms23116217

**Published:** 2022-06-01

**Authors:** Marcela K. Preininger, Daniela Kaufer

**Affiliations:** 1 Department of Integrative Biology, University of California, Berkeley, CA 94720, USA; mpreininger@berkeley.edu; 2 Department of Molecular and Cell Biology, University of California, Berkeley, CA 94720, USA; 3 Helen Wills Neuroscience Institute, University of California, Berkeley, CA 94720, USA

**Keywords:** astrocytes, senescence, blood–brain barrier, TGF beta 1, albumin, neuroinflammation

## Abstract

As the most abundant cell types in the brain, astrocytes form a tissue-wide signaling network that is responsible for maintaining brain homeostasis and regulating various brain activities. Here, we review some of the essential functions that astrocytes perform in supporting neurons, modulating the immune response, and regulating and maintaining the blood–brain barrier (BBB). Given their importance in brain health, it follows that astrocyte dysfunction has detrimental effects. Indeed, dysfunctional astrocytes are implicated in age-related neuropathology and participate in the onset and progression of neurodegenerative diseases. Here, we review two mechanisms by which astrocytes mediate neuropathology in the aging brain. First, age-associated blood–brain barrier dysfunction (BBBD) causes the hyperactivation of TGFβ signaling in astrocytes, which elicits a pro-inflammatory and epileptogenic phenotype. Over time, BBBD-associated astrocyte dysfunction results in hippocampal and cortical neural hyperexcitability and cognitive deficits. Second, senescent astrocytes accumulate in the brain with age and exhibit a decreased functional capacity and the secretion of senescent-associated secretory phenotype (SASP) factors, which contribute to neuroinflammation and neurotoxicity. Both BBBD and senescence progressively increase during aging and are associated with increased risk of neurodegenerative disease, but the relationship between the two has not yet been established. Thus, we discuss the potential relationship between BBBD, TGFβ hyperactivation, and senescence with respect to astrocytes in the context of aging and disease and identify future areas of investigation in the field.

## 1. Introduction

Age-onset neurodegenerative disorders such as Alzheimer’s disease (AD) and related dementias (ADRD) are debilitating conditions that progressively impair the memory, cognition, and daily functioning of afflicted individuals. As the average lifespan has increased in developed nations, so has the prevalence of symptomatic AD. An estimated 6.2 million adults aged 65 and older are living with Alzheimer’s dementia, presenting a substantial societal burden [1]. However, despite decades of research, negligible progress has been made in developing effective disease-modifying treatments capable of halting or reversing AD progression and associated cognitive decline. The unsatisfactory clinical trial outcomes of experimental AD therapies indicate the need for a greater understanding of the age-related processes that drive neurodegenerative diseases.

In recent years, increased focus has been placed on understanding the role of astrocytes in driving such neurological disorders. As the most abundant cell type in the adult brain, astrocytes form a sophisticated tissue-wide signaling network responsible for maintaining brain homeostasis and regulating various brain activities. Consequently, there are many potential mechanisms through which astrocytes may contribute to neural deficits in the context of advanced age and disease. Two such mechanisms are blood–brain barrier dysfunction (BBBD) and cellular senescence. Both progressively increase during aging and are associated with an increased risk of neurodegenerative disease, but the relationship between the two, if any, has not yet been established. Here, we discuss the causes and consequences of BBBD and senescence with respect to astrocytes and identify future areas of investigation in the field.

## 2. Astrocyte Functions

Astrocytes are stellated glial cells that interface with nearly every functional element of the brain. They are the most abundant glial cells in the brain and can comprise up to 50% of the tissue volume in some regions [2]. Their branch-like processes surround neurons, axons, synapses, and blood vessels, and perform numerous functions that are essential for brain homeostasis and neural functioning [3]. Therefore, astrocytes play a pivotal role in preserving brain health, as well as driving pathogenic processes [4,5]. Our understanding of astrocyte functions is continually growing as researchers study this heterogenous population of cells under different conditions, and it is increasingly evident that astrocytes perform unique functions, depending on their temporal and regional location within the brain [6,7,8,9]. Here, we briefly review the astrocyte functions related to neuronal support, immune modulation, and regulation of the blood–brain barrier (BBB), as three factors that go awry in aging and disease.

### 2.1. Neuronal Support

At the macrostructural level, astrocytes help form a functional glial network that extends from the ependyma to the pial surface via gap junctions [10,11]. The perivascular feet of astrocytes associate with the parenchymal basal lamina to form a plexus called the glia limitans. This thin, but dense, structure surrounds the pia mater, subpial space, and perivascular spaces, and plays an essential role in controlling the movement of substances from the blood or cerebrospinal fluid (CSF) into the brain parenchyma, where neurons are located [12].

In addition to their role in barrier function, astrocytes maintain the optimal conditions required for neurotransmission within the cerebral microenvironment. To transmit a signal, neurotransmitters are released from an axon terminal into the synaptic cleft, where they interact with post-synaptic receptors. Ending the transmission requires neurotransmitter uptake from the synaptic cleft by neurons and astrocytes. While neurons primarily uptake the inhibitory transmitter, gamma-amino butyric acid (GABA), astrocytes are responsible for the uptake and metabolism of the excitatory amino acid, glutamate [13,14,15]. Additionally, the propagation of nerve impulses involves cellular depolarization, which causes local extracellular changes in ion concentration. Astrocytes contain ion channels, enzymes, and receptors that enable them to modify extracellular ion concentrations and pH following depolarization to restore the surrounding milieu to its resting state [16,17,18,19]. Astrocytes are also critical in removing harmful metabolites and waste products from the brain. They can directly metabolize some soluble waste products, such as ammonia; alternatively, they collect and shuttle unwanted metabolites and soluble proteins, such as amyloid beta (Aβ) to the vasculature for elimination via the glymphatic system [15,20,21].

In addition to maintaining the cerebral microenvironment, astrocytes also participate in neurotransmission and are critical in shaping the complex circuitry of the brain [22]. Astrocytes form tripartite complexes with presynaptic and postsynaptic nerve terminals, through which they help define synaptic connections. Astrocytes are the major source of extracellular matrix proteins, cell adhesion molecules, and neurotrophic factors in the central nervous system (CNS), which are essential in promoting neurite growth and elongation [23,24,25,26,27]. Additionally, astrocytes physically associate with neuronal synapses via perisynaptic astrocytic processes (PAPs) that regulate many aspects of their formation, maturation, and function [22,28]. For example, astrocytes regulate synaptogenesis via the secretion of thrombospondins (TSP) [29] and TGFβ1 [30,31], and can specifically control the maturation and plasticity of certain circuits via the secretion of Hevin and SPARC [32,33].

### 2.2. Immune Modulation

Compared to other tissues, the brain is a relatively immune-privileged site because it lacks a significant resident lymphoid population, and the BBB substantially restricts the entry of circulating immune cells into the CNS. Astrocytes are not immune cells per se, but are capable of many immune functions, including phagocytosis, antigen presentation, and facilitating immune-cell trafficking [34,35]. Therefore, astrocytes may produce pro- or anti-inflammatory cytokines, such as IL-1β, IL-6, TNF, IL-10, IL-27, and TGF-β in response to disease, stress, or injury [34,36]. Within the CNS, they communicate bidirectionally with microglia to coordinate defense responses [37]. Along with microglia, astrocytes phagocytose neuronal material including synapses, apoptotic neurons, and degenerating axons, as well as toxic proteins, such as Aβ plaques in AD and α-synuclein in Parkinson’s disease [38,39]. Consistent with their ability to present antigens, astrocytes express major histocompatibility complex (MHC) antigens that are upregulated in response to disease [40]. For example, high levels of astrocytic MHC-II were found in the brains of patients with Parkinson’s disease (PD), which correlated with the load of pathological, phosphorylated alpha synuclein (αSYN) [41]. Notably, perivascular and infiltrated CD4+ T cells were surrounded by MHC-II expressing astrocytes, indicating astrocyte–T-cell cross-talk in the PD brain [41]. Astrocytic MHC-I is upregulated during aging, which appears to be protective, since it is associated with preserved cognitive function [42].

Due to their proximity to blood vessels, astrocytes play a critical role in mediating reciprocal communication between CNS-resident cells and the immune system. Depending on the subsets involved, astrocytes respond to T-cell signals to either boost or limit CNS inflammation. For instance, pathogenic Th17 cells signal to astrocytes via GM-CSF and IL-17 to boost neurotoxic astrogliosis [43,44,45]; however, FOXP3+ regulatory T cells secrete amphiregulin to suppress astrogliosis and promote recovery after ischemic stroke [46]. Conversely, they can also produce chemokines, such as CCL2, CXCL10, and CXCL12, which are involved in leukocyte recruitment into the CNS [47]. During aging, there is an increase in the production of astrocytic CXCL10, which serves as a chemoattractant for peripheral immune cells and aids in T-cell adhesion to endothelial cells [48]. In summary, astrocytes are critical regulators of immune responses in the CNS, as they may promote or dampen neuronal damage and inflammation, depending on the context and stimuli.

### 2.3. Blood–Brain Barrier Regulation and Maintenance

The BBB is a highly selective semipermeable barrier that restricts the entry of blood cells and plasma components into the brain parenchyma, facilitates the influx of essential nutrients, and mediates the efflux of neurotoxic products. Together, these tasks maintain an optimal environment for neuronal survival and function. The anatomical BBB consists of a continuous monolayer of endothelial cells (ECs) connected by tight junctions (TJ) and adherens junctions (containing claudin, occludin, and zonula occludens proteins). TJ proteins restrict paracellular permeability and also segregate the apical and basal domains of the cell membrane, which enables endothelial polarization [49]. The regulation of the BBB is accomplished by the neurovascular unit (NVU), a multicellular unit that functionally connects the brain and the cerebral vasculature. The NVU is composed of specialized ECs, pericytes, and perivascular astrocytes, whose end-feet sheath all cerebral blood vessels [50].

In addition to the paracellular pathway, several transcellular pathways across the BBB are carefully regulated by the NVU. Due to large surface areas of the lipophilic membranes in ECs, small gaseous molecules, such as O_2_ and CO_2_, and small lipid-soluble agents can diffuse freely though the endothelium. Specialized EC transporter proteins, such as glucose transporter 1 (GLUT1) and L-type amino acid transporter 1 (LAT1), supply the brain with glucose and amino acids, respectively. Additional transporters supply the brain with nucleosides, nucleobases, and other substances [51]. Some transporters, such as P-glycoprotein (Pgp), are energy-dependent and act as efflux transporters for neurotoxic molecules [52]. Other proteins, such as insulin and transferrin, are taken up by ECs via receptor-mediated endocytosis and then transported across the BBB in vesicles, in a process called receptor-mediated transcytosis (RMT) [53]. Native plasma proteins, such as albumin, are typically excluded from the healthy adult brain, but can cross the BBB via adsorptive-mediated transcytosis (AMT) under specific conditions [54]. AMT is also vesicle-mediated, but it involves nonspecific binding to the membrane surface charges before internalization and transport through EC cell bodies.

The perivascular end feet of astrocytes show several specialized features, including a high density of K+ transporters and aquaporin (AQP4) water channels, which are involved in ion recycling and brain-volume regulation, respectively [55]. Through a combination of cell–cell interactions and soluble factors, perivascular astrocytes regulate the expression of TJ proteins and directly modify the transport properties of the cerebral endothelium [55,56,57]. Compared with ECs cultured alone, ECs co-cultured with astrocytes or astrocyte-conditioned media were found to exhibit increased TJ formation, transporter expression, and overall improved barrier function [58]. Subsequent studies have identified the molecular mechanisms responsible for the astrocytic regulation of the BBB. For example, the astrocyte secretion of factors such as angiopoietin 1 (ANG1) and sonic hedgehog (SHH) cause ECs to upregulate TJ proteins, thus enhancing barrier tightness [59,60,61].

Beyond BBB regulation, a recent study showed that astrocytes perform a necessary and nonredundant function in adult BBB maintenance [62]. The study used a genetic DTA ablation system to conditionally and selectively ablate astrocytes from the adult mouse brain and observed significant BBBD, indicated by the leakage of cadaverine (~900 Da) and fibrinogen (340 kDa) into the parenchyma. The blood vessels within the regions of astrocyte loss had a lower expression of the TJ protein zonula occludens-1 (ZO-1), while the expression of the endothelial transporter GLUT1 remained undisturbed. BBBD persisted for several weeks following ablation, suggesting a lack of barrier repair [62].

Other roles of astrocytes in BBB function have been studied in the context of disease. For example, the E4 variant of apolipoprotein E (APOE), the main susceptibility gene for AD, leads to accelerated BBBD and cognitive decline in humans and animals [63,64]. APOE is primarily synthesized and secreted in the CNS by astrocytes and is required for BBB formation and maintenance [65]. A recent study used allele-specific knock-in mice with the human E4, E3, and E2 APOE variants and showed that the humanized APOE4, but not APOE2 or APOE3, mice exhibited BBBD, increased matrix metalloproteinases-9 (MMP9), impaired TJs, and reduced the astrocyte end-foot coverage of blood vessels [66]. This is a seminal example of how astrocyte dysfunction can directly lead to BBBD and subsequent neurological disease. Additional mechanisms through which astrocytes contribute to BBBD in aging and disease continue to be explored.

## 3. Mechanisms of Astrocyte-Mediated Neuropathology in the Aging Brain

Brain aging consists of a set of physiological and cellular changes that result in reduced neural function. The salient changes associated with aging include significant decreases in certain neuronal populations, dendritic spines, axonal arborization, post-synaptic density, and cortical volume [67]. Together, these physiological changes result in cognitive impairment and memory loss. Given the many diverse functions astrocytes perform in the brain, it is logical that astrocyte dysfunction would result in neural deficits and neuropathology. Thus, there is substantial interest in understanding the precise role of astrocytes in age-related neural dysfunction, especially in neurodegenerative diseases.

Several molecular changes have been observed in aging astrocytes that may negatively affect neurons, including the activation of complement system genes and increased oxidative stress. Aged astrocytes express C3 and C4B complement component genes [68], which may impair plasticity by causing structural changes in neurons [69]. Furthermore, oxidative stress increases during aging and leads to the intracellular accumulation of reactive oxygen species (ROS). In turn, this causes calcium overload in astrocytes, which is associated with hippocampal neuronal dysfunction [70]. While these pathways are relevant, this review focuses on two other critically important and potentially related mechanisms through which astrocytes contribute to neuropathology in aging: blood–brain barrier dysfunction (BBBD) and cellular senescence.

### 3.1. TGFβ Hyperactivation following Blood–Brain Barrier Dysfunction (BBBD)

One of the physiological consequences of advanced age is the progressive decline in NVU function, which results in increased BBB permeability and the leakage of blood-borne molecules into the neural microenvironment. Clinical studies have shown that BBBD is prevalent in aging individuals [71,72,73] and is correlated with neurodegenerative diseases, especially ADRD [74,75,76,77]. The BBBD phenotype is multifaceted and adversely affects nearly every element of the brain. The murine endothelium in BBBD is characterized by the decreased expression of TJ proteins [78,79], increased EC pinocytosis [80,81], and the decreased expression of the GLUT1 transporter [82]. In humans, BBBD is characterized by focal endothelium degeneration [83] and the decreased EC expression of the Pgp transporter [84,85]. The decreased GLUT1 expression in BBBD impairs the influx of glucose, and decreased Pgp expression impairs the efflux of neurotoxic molecules, such as Aβ, allowing them to accumulate in the brain tissue, where they contribute to inflammation and neural degeneration [84,86,87]. In humans and mice, pericytes and astrocytes have reduced vascular coverage in BBBD, and astrocytes exhibit the downregulation of AQP4, the protein primarily responsible for end-foot adhesion and polarization at the vascular wall [88,89,90,91,92]. The leakage of neurotoxic blood-derived proteins into the parenchyma triggers microglial activation and astrogliosis as an injury response, contributing to neuroinflammation [93,94,95]. Studies in mice have shown that in the short term, this inflammation triggers microglia to migrate to damaged vessels and maintain the BBB via the expression of the TJ protein, Claudin-5; however, during sustained inflammation, microglia phagocytose astrocytic end-feet, further impairing BBB integrity [96]. In addition to the leakage caused by the loss of BBB integrity, recent evidence suggests that there is a global age-related shift in BBB transcytosis mechanisms that further increases the influx of neurotoxic proteins such as albumin, fibrinogen, and autoantibodies into the aging brain [92]. Specifically, the aging BBB exhibits diminished RMT transport and an increased non-specific transport of macromolecules compared to young brains. Regardless of the mechanism of entry, the dysregulated presence of serum proteins causes astrogliosis and neuroinflammation, and the dysfunctional phenotype of the NVU components in BBBD creates a hostile environment for neurons. As a result, white matter, neuronal axons, and synapses are compromised in BBBD, leading to cognitive impairment [83].

The hippocampus, the brain region’s center for learning and memory, appears to be especially vulnerable to age-related BBBD. Hippocampal BBBD is associated with mild cognitive impairment (MCI) and occurs before tissue atrophy and dementia in AD patients [71,97]. Notably, hippocampal neural hyperexcitability is also an early biomarker of MCI in humans that precedes dementia [98,99] and is associated with disease progression in rodent AD models [100,101]. These findings suggest there is a relationship between BBBD and neural hyperexcitability in aging. Indeed, a recent study from our group identified astrocytic TGFβ signaling as a mechanistic link between BBBD and hyperexcitability in the aging hippocampus and established how these factors contribute to cognitive impairment in humans and mice [73,102]. This study was based on the observation that the context and symptomatology of age-related neural dysfunction resemble those observed in traumatic brain injury (TBI). Both aging and TBI involve a period of BBBD followed by secondary neural dysfunction and are associated with an increased risk of neurodegenerative disease and dementia [74,103]. In both cases, the neural dysfunction is characterized by an excitation–inhibition (E/I) imbalance, hippocampal and cortical circuit hyperexcitability, and cognitive deficits [73,102].

TBI causes sudden and pronounced BBBD in patients, which can persist for months to years after the initial injury [104,105,106]. Studies in mice and rats have shown that BBBD induced by deoxycholic acid sodium salt (DOC) or direct injury enables blood-derived serum albumin and fibrinogen molecules to extravasate into the brain, where they elicit a robust inflammatory response mediated by astrocytic TGFβ signaling [107,108,109]. Albumin binds to the type II TGFβ receptor (TGFβR) on astrocytes and activates the canonical ALK5 type I TGFβR after receptor dimerization. This results in the phosphorylation of the downstream effector protein SMAD2 (pSMAD2) and the subsequent activation of pro-inflammatory and epileptogenic transcriptional programs [110,111,112]. The molecular hallmarks of this altered astrocytic phenotype are the upregulation of the cytoskeletal intermediate filaments, GFAP and vimentin, as well as S100 calcium-binding proteins. In mouse and rat models of BBBD, the astrocytic inflammatory program involves the production of cytokines, such as interleukin 6 (IL-6), monocyte chemoattractant protein-1 (MCP-1; CCL2), and matrix metalloproteinase 9 (MMP-9), which degrades critical extracellular structures and destabilizes perineuronal nets (PNNs) around inhibitory interneurons [107,111]. In rodent models, the astrocytic epileptogenic program features the upregulation of excitatory glutamate receptors, the activation of complement components C1 and C2, and the downregulation of voltage-gated potassium channels (Kir4.1), plasticity-associated NMDA receptors, and genes involved in inhibitory GABAergic transmission [107,111]. Notably, albumin-induced TGFβ activation itself upregulates the astrocytic expression of TGFβ and its receptors, resulting in a positive feedback loop that can cause the altered phenotype to persist [73,110]. In this state, the normal homeostatic neurotransmitter recycling and ion buffering functions of astrocytes are impaired, leading to disrupted neural communication. The remodeling of neural circuits occurs over time, which causes network hyperexcitability, E/I imbalance, and cognitive deficits [108,112,113,114,115]. In mice, the astrocytic secretion of TGFβ was shown to increase the neuronal expression of c1q [116], a complement protein that mediates synapse elimination [117] and increases in the CNS with age [118].

In the case of aging, the onset of BBBD is more gradual than in TBI, but it is also temporally constrained. Depending on an individual’s lifestyle and risk factors, BBBD begins as early as middle age in the human hippocampus and progresses in scope and severity into advanced age [71,73,75,76]. Interestingly, our study found that albumin-induced astrocytic TGFβ activation is a mechanism underpinning key neural deficits in aging [73]. In mice, immunohistochemistry (IHC) revealed that albumin begins to accumulate in hippocampal astrocytes as early as 12 months (“middle age”) and is consistently elevated in aging up to the end of life at ~2 years, indicating BBBD. Concurrent with the time course of BBBD, aged mice showed a progressive increase in the amount of pSMAD2 colocalized with albumin-positive hippocampal astrocytes, indicating BBBD-induced TGFβ signaling activation. Additionally, a Western blot analysis showed an increased concentration of active TGFβ1 (a positive feedback output of the TGFβ pathway) in the hippocampi of aged mice. Compared to the young mice, the aged mice exhibited increased seizure vulnerability and paroxysmal slow-wave events (PSWEs), indicating increased neural hyperexcitability. The activation of TGFβ signaling via serum albumin infusion into the cerebral ventricles of young mice was sufficient to elicit increased hyperexcitability, aberrant neural activity, and learning impairment, as measured by the Morris Water Maze assay. The pharmacological inhibition of the ALK5 type I TGFβR with the small molecule drug IPW-5371 significantly reduced TGFβ activation and seizure vulnerability in young mice infused with albumin. Importantly, the conditional knockdown of the type II astrocytic TGFβR and systemic treatment with IPW were both able to reverse the BBBD-induced neuropathological changes observed in aged mice, providing further evidence for the mechanistic role of TGFβ activation in driving these changes [73,102].

### 3.2. Cellular Senescence

Another consequence of advanced age is the accumulation of senescent cells [119,120,121,122,123,124]. Cellular senescence is an important anti-cancer mechanism that occurs in replication-competent cell types. The senescence response permanently arrests cell growth in response to stresses that can promote malignant transformation [125]. There are two main types of senescence: replicative senescence is triggered by extreme telomere erosion caused by repeated cell division [126], while stress-induced premature senescence (SIPS) is triggered by sublethal levels of cellular stress in the absence of telomere erosion. A common trigger of SIPS is DNA damage, which can be caused by factors such as environmental toxins, viral infection, and ionizing radiation [127,128,129]. Other stressors can trigger SIPS by activating molecular pathways that are associated with cellular transformation. For example, epigenetic factors, mitochondrial dysfunction, disrupted nutrient signaling, chronic inflammation, oxidative stress, and proteotoxic stress can also cause SIPS [130]. The senescence response ultimately activates the p53/p21^WAF1^ and p16^INK4A^/pRB tumor suppressor pathways, which establish and maintain growth arrest [127,131,132,133]. The senescent phenotype is not precisely defined and differs among cell types and conditions but is generally characterized by permanent cell-cycle arrest, increased lysosomal mass, the loss of nuclear integrity, decreased functional capacity, and the expression of the senescence-associated secretory phenotype (SASP) [119,134,135,136,137].

Senescent cells accumulate with age in a variety of tissues and contribute to age-associated tissue dysfunction and disease. A seminal study in the field showed that senescent cells accumulate in a progeroid mouse model of accelerated aging, and that the elimination of senescent cells via a drug-inducible transgene extends lifespan and prevents the onset of aging phenotypes, including cataracts, sarcopenia, and subcutaneous fat loss [138]. There are two known mechanisms through which senescent cells drive tissue aging and dysfunction. The first is through loss of function, in which senescent cells have a reduced ability to perform their vital activities within t tissues. The second is through the SASP, in which senescent cells secrete inflammatory cytokines, chemokines, growth factors, and proteases to communicate cellular damage to surrounding cells [130,139,140]. Many SASP factors are considered beneficial in limited quantities because they stimulate tissue repair and increase immune surveillance; however, their abundance in aging tissue has been shown to impair tissue structure and function by disrupting intercellular signaling and maintaining a pro-inflammatory environment [139,141,142,143,144].

While normally quiescent in the adult brain, astrocytes maintain their ability to replicate, and are thus susceptible to SIPS [145]. Astrocytic SIPS has been observed in response to oxidative stress [146], proteasome inhibition [146], HIV infection [147], methamphetamine [147], Aβ oligomers [148], and the environmental toxin, paraquat [149]. Human and animal studies have demonstrated that senescent cells accumulate in the aging CNS and are associated with neuroinflammation and neurodegeneration [150,151,152,153,154]. In human brain samples, the prevalence of senescent astrocytes in the brain was shown to increase with age, with a significantly higher burden observed in the cortices of AD patients compared to age-matched controls [148]. Recent studies in animal models have demonstrated a causal relationship between glial senescence and neurodegeneration. The accumulation of senescent astrocytes and microglia was found in the cortices and hippocampi of a mouse model of tau-dependent pathology, and the genetic ablation of these cells reduced tau deposition and prevented neuronal degeneration [155]. Similarly, senescent astrocytes were found in abundance in the substantia nigra of a mouse model of paraquat-induced Parkinson’s disease, and their selective genetic ablation was sufficient to reduce nigral dopaminergic cell loss and improve motor function [149]. Notably, novel pharmacological agents have been developed that can selectively eliminate senescent cells, and they are being explored in clinical trials for the treatment of age-related diseases [156,157,158]. These findings indicate that senescent astrocytes actively participate in driving age-associated neural dysfunction and pathology, including neurodegeneration, and can be leveraged as therapeutic targets.

As with other senescent cell types, senescent astrocytes appear to drive neural dysfunction via both loss of function and their SASP. Given the important role of astrocytes in supporting brain homeostasis and maintaining the neural microenvironment, the senescence-associated loss of astrocyte function is especially detrimental to neuronal survival and performance [159]. Indeed, substantial impairments have been reported in rat astrocyte functions cultured in vitro, including impaired wound healing ability, phagocytic uptake, metabolic function, and neuroprotective capacity [160,161]. Furthermore, the downregulation of potassium (Kir4.1) and glutamate (EAAT1/2) transporters in human senescent astrocytes promotes neurotoxicity in cortical neurons [162]. As a heterogenous population, it is likely that other specialized astrocyte functions are also impaired that have not yet been identified. Additionally, the astrocytic SASP participates in driving pathological changes in the aging brain by generating a chronic inflammatory environment. Senescent astrocytes from humans and mice secrete inflammatory and proteolytic SASP factors, such as IL-6, IL-1β, CCL2, MMP-3, and MMP-9 [148,149,155,163,164]. Interestingly, these factors are elevated in the cerebrospinal fluid (CSF) and sera of AD patients, suggesting that senescence-associated inflammation accompanies and may contribute to the progression of AD [165,166]. In particular, IL-6 is a salient SASP component and a biomarker of AD [167], whose overexpression drives neurodegeneration in AD models [168,169,170].

## 4. Perspectives on BBBD, TGFβ Signaling, and Astrocyte Senescence in Aging

As detailed above, a growing body of evidence suggests that both BBBD and astrocyte senescence progressively increase in the aging brain and are independently associated with an increased risk of neurodegenerative disease, but the relationship between the two has not been established. Given the salience of TGFβ signaling activation in aging phenotypes and neural dysfunction, BBBD-induced TGFβ hyperactivation should be explored as a potential mechanistic link between BBBD and senescence in the aging brain.

Studies show that TGFβ activation mediates senescence in various cell types in vitro, including fibroblasts [171,172,173], keratinocytes [174], mesenchymal stem cells (MSCs) [175], glioblastoma cells [176], cardiomyocytes [177], and lung epithelial cells [178]. Furthermore, TGFβ1, as a SASP component, was found to be an important mediator of paracrine senescence in vivo [179]. Furthermore, the pharmacological attenuation of age-associated TGFβ activation has rejuvenating effects in the aged brain [73,180], including the suppression of cellular senescence [181]. In astrocytes, previous reports have identified triggers of astrocyte senescence, such as oxidative stress [146], Aβ oligomers [148,182], HIV infection, and drug abuse [147]. Notably, TGFβ signaling is also implicated in these senescence mechanisms. In the context of oxidative stress, TGFβ activation increases the production of reactive oxygen species (ROS) by impairing mitochondrial function and inducing NADPH oxidases [183,184]. Indeed, TGFβ1 was found to induce the senescence of bone marrow MSCs via increased mitochondrial ROS [175]. TGFβ activation also suppresses the synthesis of antioxidant enzymes, such as glutathione, causing redox imbalance and promoting further oxidative stress [185]. In the case of Aβ pathology, astrocytic TGFβ overexpression increases Aβ generation in transgenic mice [186,187,188], and TGFβ1 expression is elevated in cortical astrocytes surrounding Aβ plaques in AD patients [189,190]. HIV infection and drug abuse were found to mediate astrocyte senescence in a β-catenin-dependent manner [147]. Notably, TGFβ1 operates through the canonical WNT/β-catenin pathway, and these two pathways stimulate each other via SMAD effector proteins [191]. The precise molecular mechanisms through which TGFβ signaling mediates senescence in astrocytes are unknown, but some evidence suggests that epigenetic alterations may be involved. A recent study found that canonical TGF-β/SMAD signaling promotes SIPS in cardiomyocytes via the miR-29-induced loss of H4K20me3, and that disrupting TGFβ signaling improves cardiac function in aged mice [177].

Together, these findings indicate that TGFβ activation is a salient factor in age-related pathological changes and can directly mediate the senescence response under certain conditions. Indeed, the results from a recent study suggest that BBBD-induced TGFβ activation via albumin extravasation into the brain parenchyma is a potential physiological trigger of astrocyte senescence [192]. This study utilized an animal model of BBBD in which albumin was continuously infused into the lateral ventricles of adult mice. One week of albumin infusion significantly increased TGFβ signaling activation and the burden of senescent astrocytes in hippocampal tissue. The pharmacological inhibition of TGFβR ALK5 or the conditional genetic knockdown of astrocytic TGFβR prior to albumin infusion were sufficient to prevent albumin-induced astrocyte senescence. In addition to activating canonical TGFβ signaling, albumin uptake activates the p38MAPK pathway in astrocytes [94], and it is known to play a central role in the regulation of astrocyte senescence and SASP expression [148,193]. Thus, it follows that the simultaneous activation of canonical TGFβ, p38MAPK, and, potentially, other unknown pathways by albumin can mediate astrocyte senescence.

Reciprocally, the molecular and functional effects of senescent astrocytes on BBB integrity have not been established. Certainly, astrocytes produce SASP factors, such as MMPs and IL-6, which are known to increase endothelial barrier permeability. MMPs induce BBBD via the disruption of TJ proteins [194,195], and IL-6 upregulates cell adhesion molecules (CAMs) on ECs, which facilitates leukocyte transmigration across the endothelium. Interestingly, one study found that the accumulation of senescent ECs and pericytes is associated with compromised BBB integrity due to reduced TJs; however, the blood vessel coverage by astrocytic end-feet was not altered [196]. This suggests that EC and pericyte senescence is not the proximal cause of the reduced astrocyte end-foot coverage observed in age-associated BBBD. These findings raise another possibility—that each cellular component of the NVU is differentially susceptible to physiological triggers of senescence. Indeed, TGFβ signaling is complex and context-dependent [197,198]; therefore, TGFβ hyperactivation may have variable effects in different cell types and conditions. Future studies should seek to elucidate the context in which various triggers promote senescence during aging and the extent of their respective contributions.

## 5. Concluding Remarks and Future Directions

Astrocytes are a diverse population of cells that perform essential functions in the brain related to neuronal support, immune modulation, and blood–brain barrier regulation and maintenance. Collectively, these functions ensure that the brain environment remains optimal for neuronal function. In aging, astrocytes adopt a neurotoxic phenotype following age-related BBBD that is mediated by TGFβ hyperactivation. Furthermore, aging is associated with an accumulation of senescent astrocytes, which contribute to neuroinflammation and neurodegeneration. Given that TGFβ is a salient factor in aging pathologies and is known to mediate senescence in various cell types, BBBD-induced TGFβ activation via blood protein extravasation should be further explored as a potential trigger of astrocyte senescence in the aging brain. Identifying the physiological triggers of astrocyte senescence is important for understanding the complex series of events that facilitates the development of neurodegenerative disease.

Astrocytes play an important role in BBB regulation and maintenance through their involvement in the NVU. Since the NVU is highly interdependent, the impacts of perivascular astrocyte senescence on each component of the NVU merit investigation. Genetic tools that enable the selective ablation of senescent astrocytes [149] should be leveraged to investigate the role of senescent astrocytes in the context of BBBD. Another unanswered question in the context of aging is how the global shift in transcytosis mechanisms manifests in each NVU cell type. Specifically, how do perivascular astrocytes experience this shift, and does cellular senescence play a role in facilitating this shift? In other words, do senescent cells experience more nonspecific transcytosis than their youthful counterparts?

**Figure 1:**
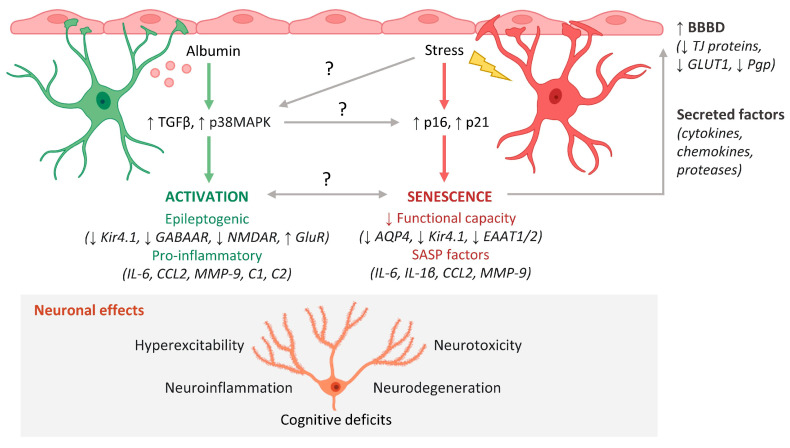
Mechanisms of astrocyte-mediated neuropathology in the aging brain. Blood–brain barrier dysfunction (BBBD) and cellular senescence progressively increase during aging and are associated with an increased risk of neurodegenerative disease. Two processes that result in dysfunctional astrocyte phenotypes and subsequent neuropathology are BBBD-associated astrocyte activation and astrocyte senescence. BBBD causes albumin to leak into the brain, where it activates TGFβ and p38MAPK signaling in astrocytes, which elicits an epileptogenic and pro-inflammatory astrocyte phenotype. The epileptogenic phenotype is characterized by downregulation of K+ transporters (Kir4.1), inhibitory GABA_A_ receptors (GABAAR), and NMDA receptors (NMDAR) and upregulation of excitatory glutamate receptors (GluR). Second, certain types of cellular stress cause expression of tumor suppressors p16 and p21, which elicit a senescent phenotype characterized by decreased functional capacity and SASP expression. This decreased capacity involves downregulation of aquaporin 4 (AQP4), K+ transporters (Kir4.1), and glutamate transporters (EAAT1/2). Factors such as cytokines, chemokines, and proteases secreted by activated and senescent astrocytes may act on the endothelium to further exacerbate BBBD via downregulation of tight junction (TJ) proteins, glucose transporters (GLUT1), and waste efflux transporters (Pgp). Dysfunctional astrocytes contribute to neuronal hyperexcitability, neuroinflammation, neurotoxicity, and neurodegeneration, which manifest as cognitive deficits in the aging brain. Questions remain regarding the mechanistic links and relationships between BBBD-associated astrocyte activation and senescence. This figure was created with cell images from BioRender.

Given the importance of astrocytes in preserving the BBB, it is likely that initial BBBD-induced astrocyte senescence establishes a positive feedback loop that results in progressive neurological decline. In this scenario, the accumulation of functionally impaired senescent astrocytes causes more BBBD and, thus, more BBBD-induced astrocyte senescence—perpetuating a vicious cycle of BBBD and associated functional decline (Figure 1). Further studies should investigate how this process is regulated and explore potential strategies to prevent its onset.

## Abbreviations

AD	Alzheimer’s disease
BBB	blood-brain barrier
BBBD	blood-brain barrier dysfunction
CNS	central nervous system
EC	endothelial cells
SASP	senescence-associated secretory phenotype
MCI	mild cognitive impairment
NVU	neurovascular unit
SIPS	stress-induced premature senescence
TBI	traumatic brain injury
TJ	tight junctions
